# Supplementary material for: Mitophagy Facilitates Cytosolic Proteostasis to Preserve Cardiac Function
Source: bioRxiv. 2024 Nov 26:2024.11.24.624947. Preprint. [Version 1] doi: 10.1101/2024.11.24.624947 (PMC11623534; doi:10.1101/2024.11.24.624947)
Supplement: Supplement 1 [file NIHPP2024.11.24.624947v1-supplement-1.pdf]

bioRxiv preprint doi: https://doi.org/10.1101/2024.11.24.624947; this version posted November 26, 2024. The copyright holder for this preprint (which was not certified by peer review) is the author/funder, who has granted bioRxiv a license to display the preprint in perpetuity. It is made available under aCC-BY 4.0 International license.

SUPPLEMENTARY DATA for Mitophagy Facilitates Cytosolic Proteostasis to Preserve Cardiac Function

Supplementary Figures

Figure S1

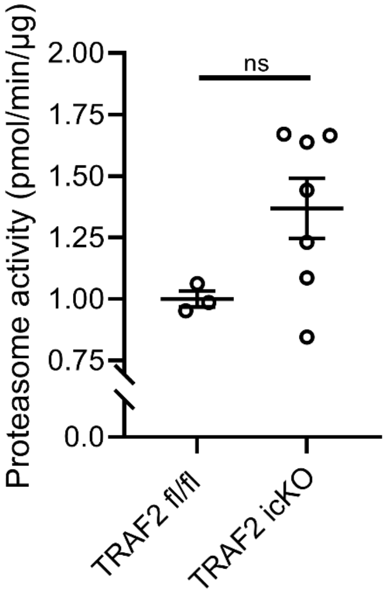

Figure S1. Cardiac myocyte deletion of TRAF2 does not decrease cardiac proteasome activity. Proteasome activity assessed in heart tissue from TRAF2-icKO mice versus TRAF2 fl/fl controls, modeled as in Figure 1A. Graph shows mean +/- SEM. No statistically significant difference was detected by t-test.

bioRxiv preprint doi: https://doi.org/10.1101/2024.11.24.624947; this version posted November 26, 2024. The copyright holder for this preprint (which was not certified by peer review) is the author/funder, who has granted bioRxiv a license to display the preprint in perpetuity. It is made available under aCC-BY 4.0 International license.

Figure S2

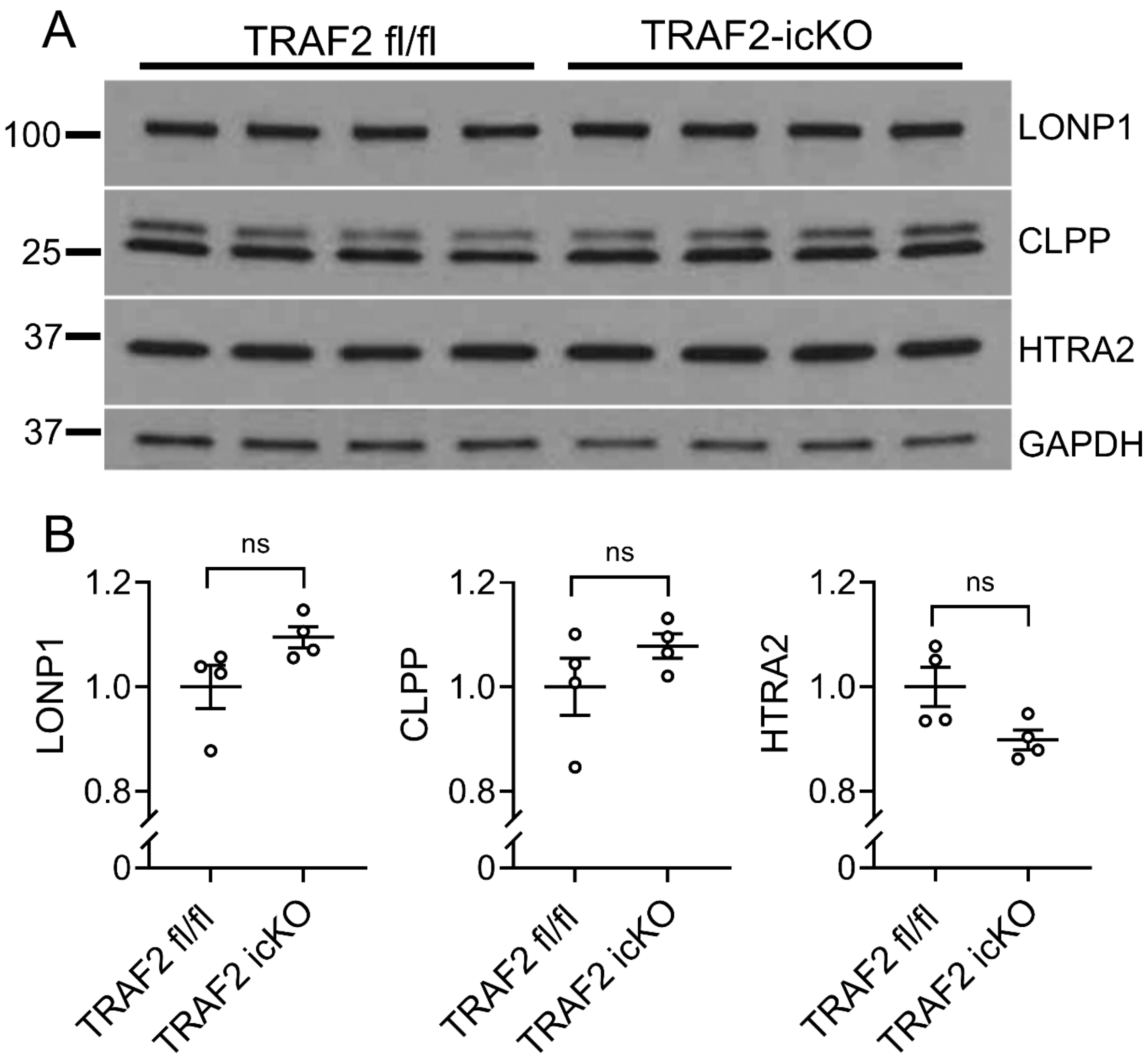

Figure S2. TRAF2 deletion does not alter mitochondrial protease levels. A Immunoblotting for mitochondrial proteases LONP1, CLPP, and HTRA2 in TRAF2-icKO and TRAF2 fl/fl hearts, modeled as in Figure 1A. **B** Quantitation of LONP1, CLPP, and HTRA2 levels from A. Levels are normalized to GAPDH as the loading control. No statistically significant differences were detected between groups by t-test.

bioRxiv preprint doi: https://doi.org/10.1101/2024.11.24.624947; this version posted November 26, 2024. The copyright holder for this preprint (which was not certified by peer review) is the author/funder, who has granted bioRxiv a license to display the preprint in perpetuity. It is made available under aCC-BY 4.0 International license.

Figure S3

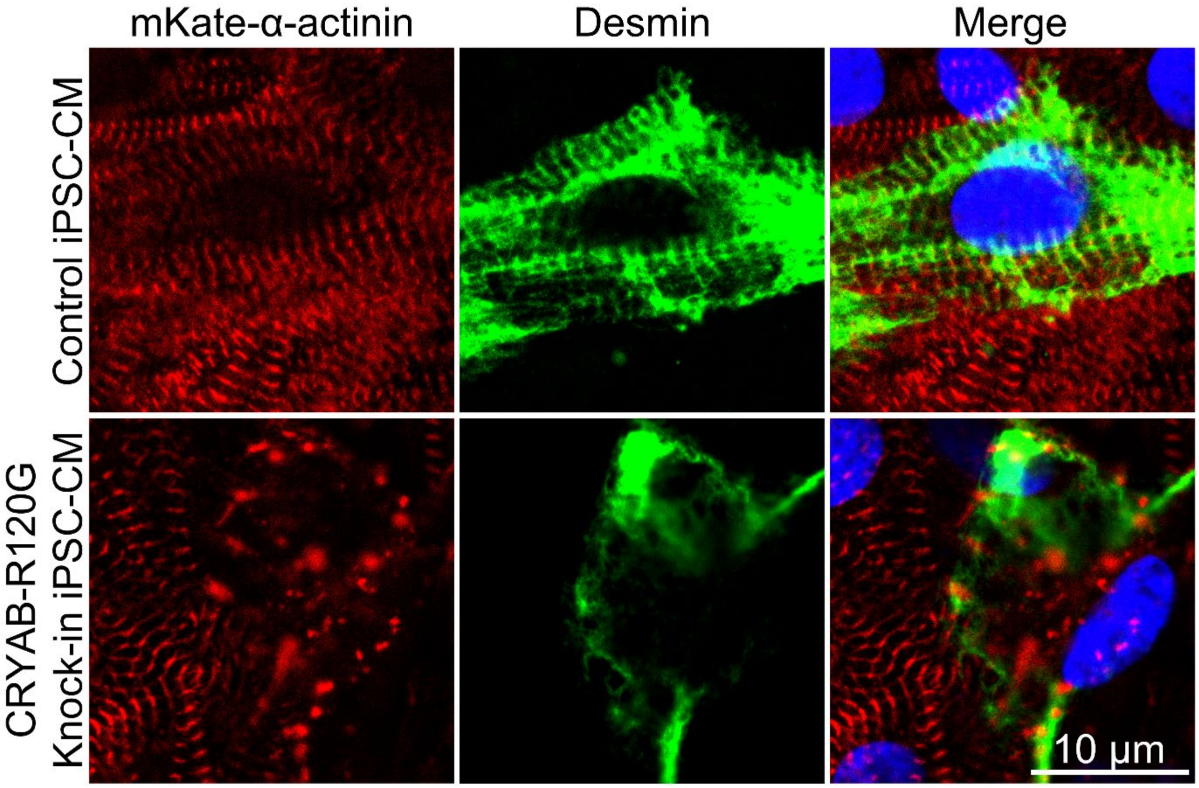

Figure S3. DESMIN aggregates are observed in R120G knock-in iPS cell derived cardiac myocytes. Representative images from induced pluripotent stem cell (iPSC)-derived cardiac myocytes (cultured in aggregates for 30 days) from iPSC lines homozygous for knock-in of R120G mutation and isogenic controls expressing mKate-tagged α-actinin and stained with antibody against DESMIN. Images demonstrate localization of DESMIN and α-actinin aggregates in R120G knock-in iPSC-derived cardiac myocytes (bottom panel), as compared with localization of these proteins in a sarcomeric pattern in control iPSC-derived cardiac myocytes (top panel). DAPI stained nuclei are blue.

bioRxiv preprint doi: https://doi.org/10.1101/2024.11.24.624947; this version posted November 26, 2024. The copyright holder for this preprint (which was not certified by peer review) is the author/funder, who has granted bioRxiv a license to display the preprint in perpetuity. It is made available under aCC-BY 4.0 International license.

Figure S4

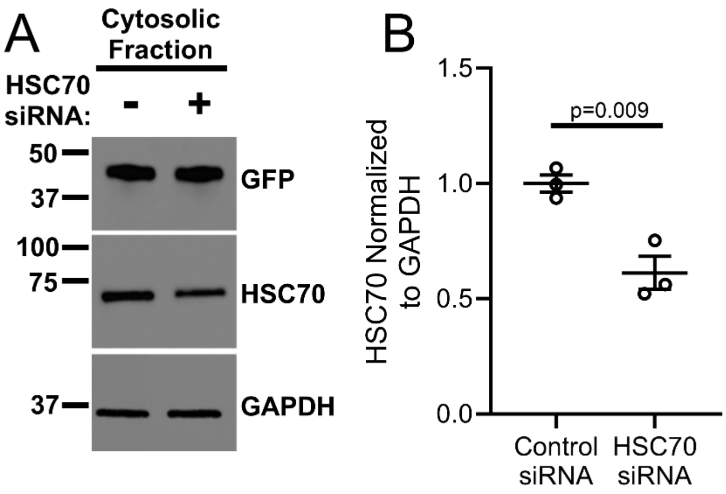

Figure S4. siRNA transfection results in knockdown of HSC70 in HEK293 cells. A, B Representative immunoblot (A) depicting expression of HSC70 and GFP in GFP-tagged R120G CRYAB transfected with siRNA targeting HSC70 (depicted as '+') or scrambled control (depicted as '-') in HEK293 cells with quantitation of HSC70 abundance (B, expressed as fold change over control). GAPDH is employed as loading control.

bioRxiv preprint doi: https://doi.org/10.1101/2024.11.24.624947; this version posted November 26, 2024. The copyright holder for this preprint (which was not certified by peer review) is the author/funder, who has granted bioRxiv a license to display the preprint in perpetuity. It is made available under aCC-BY 4.0 International license.

Figure S5

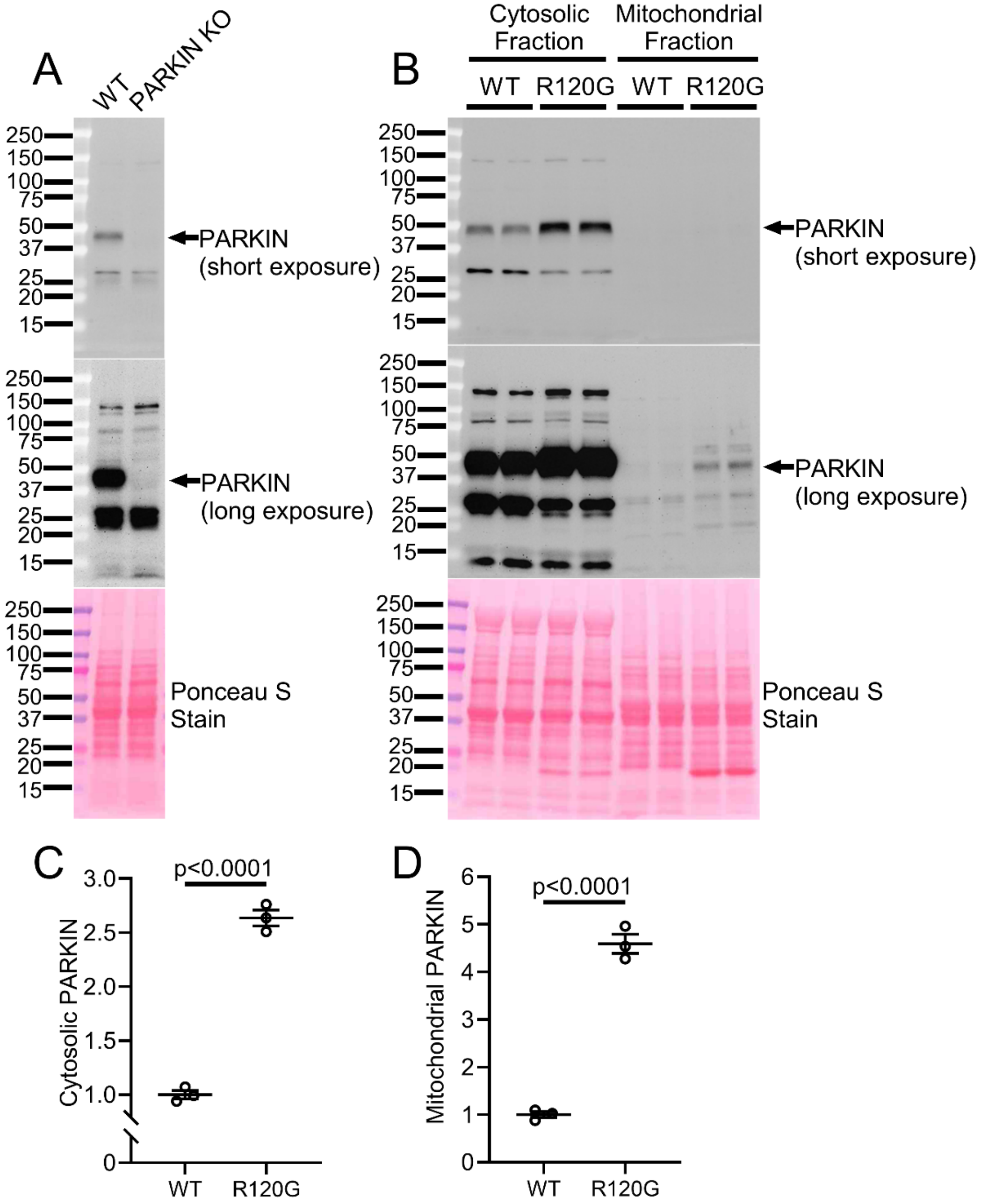

Figure S5. PARKIN expression and mitochondrial localization is increased in CRYAB-R120G hearts. A Immunoblotting in wild-type (WT) and Park2 null (PARKIN KO) heart samples demonstrating specificity of the PARKIN antibody. Arrow indicates PARKIN-specific band. **B** Immunoblotting on cytosolic and mitochondrial fractions from WT and CRYAB-R120G hearts at 20-24 weeks of age. PARKIN blots show short and long exposures. Cytosolic and mitochondrial samples are from the same fractionations shown in Figure 5E; see this panel for cytosolic (GAPDH) and mitochondrial (COXIV, VDAC) markers to demonstrate successful separation of mitochondrial and cytosolic fractions in these

bioRxiv preprint doi: https://doi.org/10.1101/2024.11.24.624947; this version posted November 26, 2024. The copyright holder for this preprint (which was not certified by peer review) is the author/funder, who has granted bioRxiv a license to display the preprint in perpetuity. It is made available under aCC-BY 4.0 International license.

samples. **C-D** Quantitation of PARKIN levels in cytosolic and mitochondrial fractions in immunoblotting in panel b. Samples are normalized to total protein as shown by Ponceau S staining. *P* values shown are by t-test.

bioRxiv preprint doi: https://doi.org/10.1101/2024.11.24.624947; this version posted November 26, 2024. The copyright holder for this preprint (which was not certified by peer review) is the author/funder, who has granted bioRxiv a license to display the preprint in perpetuity. It is made available under aCC-BY 4.0 International license.

Figure S6

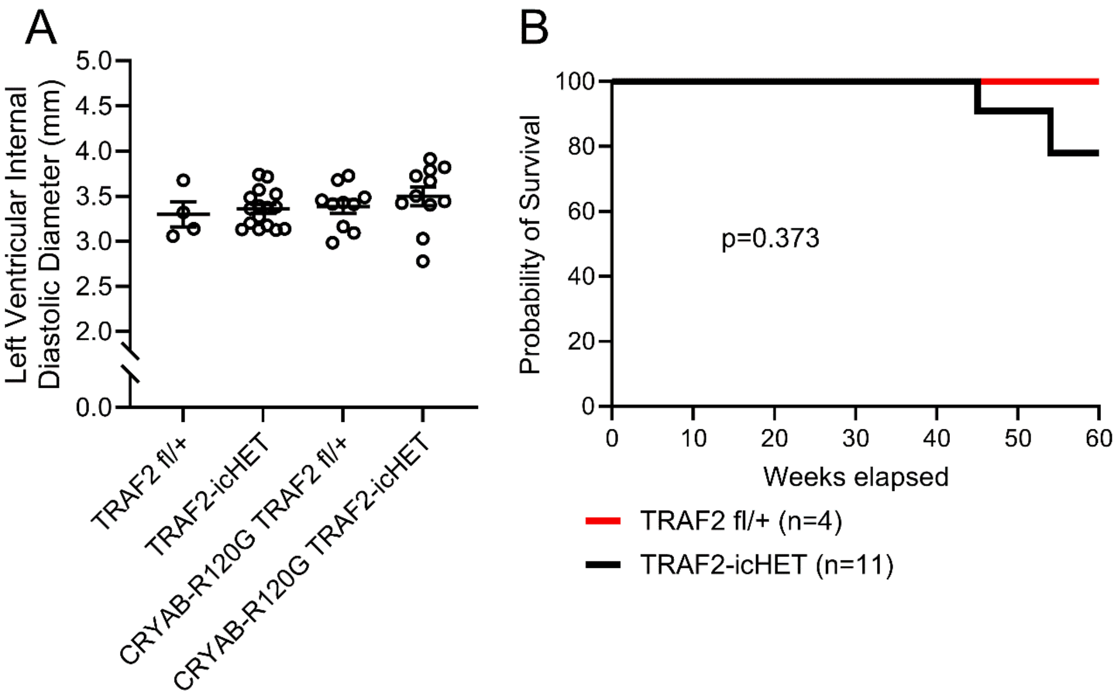

Figure S6. Reduction in cardiac myocyte TRAF2 does not affect left ventricular dimensions in Myh6-R120G CRYAB transgenic mice or alter mortality in wild type mice. A Left ventricular internal diastolic diameter by M-mode echocardiography in 20-week-old CRYAB-R120G mice with cardiac myocyte-specific inducible deletion of one *Traf2* allele (termed R120G TRAF2-icHET; see Figure 6A for experimental schematic). These mice were compared to R120G TRAF2 fl/+ mice without the MerCreMer transgene, and to TRAF2 fl/+ and TRAF2-icHET control mice without CRYAB-R120G. Statistical comparisons were performed by one-way ANOVA followed by Tukey's test for multiple comparison testing between groups. There were no statistically significant differences between groups in panel A. **B** Kaplan-Meier survival analysis of TRAF2-icHET mice and TRAF2 fl/+ controls. No statistically significant differences were detected by Mantel-Cox log-rank testing.

bioRxiv preprint doi: https://doi.org/10.1101/2024.11.24.624947; this version posted November 26, 2024. The copyright holder for this preprint (which was not certified by peer review) is the author/funder, who has granted bioRxiv a license to display the preprint in perpetuity. It is made available under aCC-BY 4.0 International license.

Figure S7

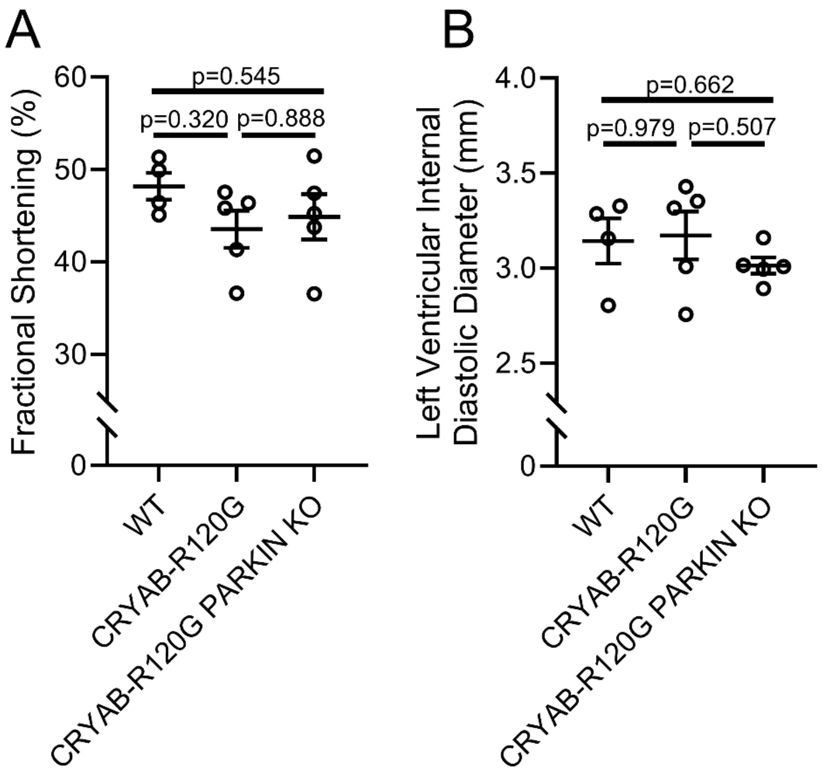

Figure S7. PARKIN ablation in Myh6-R120G CRYAB transgenic mice does not affect left ventricular structure and function. **A, B** Left ventricular endocardial fractional shortening (A) and left ventricular internal diastolic diameter (B) by M-mode echocardiography in 20-week-old CRYAB-R120G mice with and without concomitant *Park2* null alleles (as homozygous, PARKIN KO) and wild type as control. No statistically significant differences were detected by one-way ANOVA followed by Tukey's test for multiple comparison testing between groups.

bioRxiv preprint doi: https://doi.org/10.1101/2024.11.24.624947; this version posted November 26, 2024. The copyright holder for this preprint (which was not certified by peer review) is the author/funder, who has granted bioRxiv a license to display the preprint in perpetuity. It is made available under aCC-BY 4.0 International license.

Figure S8

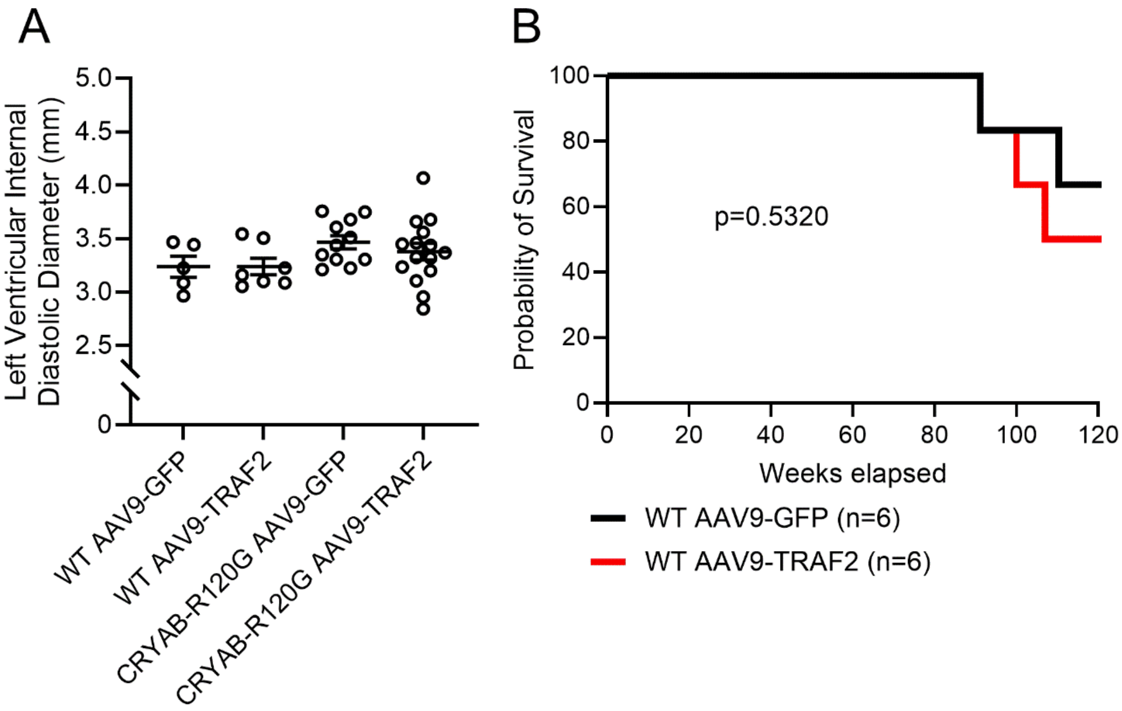

Figure S8. AAV9-mediated transduction of TRAF2 does not affect left ventricular dimension in Myh6-R120G CRYAB transgenic mice or alter mortality in wild type mice. A Left ventricular internal diastolic diameter by M-mode echocardiography in 20-week-old CRYAB-R120G mice and wild type mice with AAV9-cardiac Troponin T promoter driven transduction of TRAF2 or GFP at 8 weeks of age (See Figure 8A for experimental schematic). Statistical comparisons were performed by one-way ANOVA followed by Tukey's test for multiple comparison testing between groups. There were no statistically significant differences between groups in panel A. **B** Kaplan-Meier survival analysis of wild type mice with AAV9-cardiac Troponin T promoter driven transduction of TRAF2 or GFP at 8 weeks of age. No statistically significant differences were detected by Mantel-Cox log-rank testing.

bioRxiv preprint doi: https://doi.org/10.1101/2024.11.24.624947; this version posted November 26, 2024. The copyright holder for this preprint (which was not certified by peer review) is the author/funder, who has granted bioRxiv a license to display the preprint in perpetuity. It is made available under aCC-BY 4.0 International license.

Figure S9

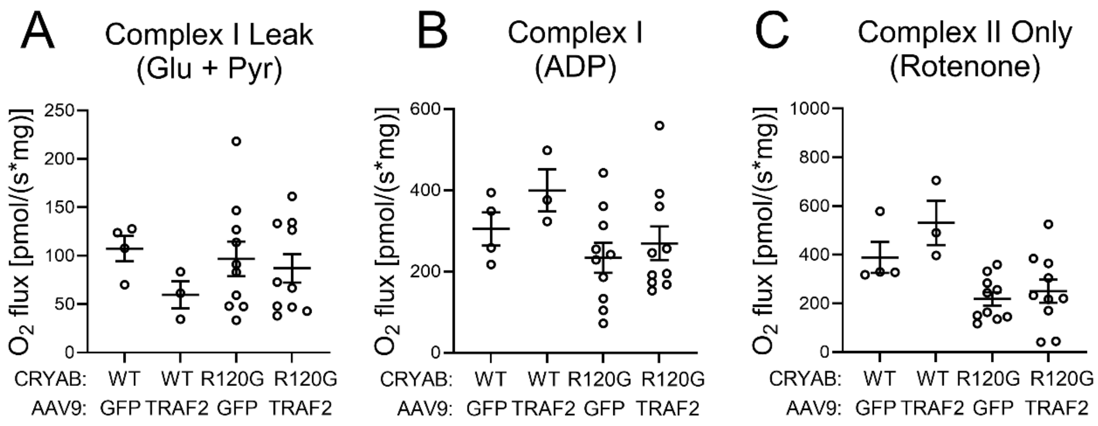

Figure S9. High resolution respirometry in Myh6-R120G CRYAB transgenic mice transduced via AAV9-GFP or AAV9-TRAF2. A-C. High-resolution respirometry was performed to measure oxygen consumption [volume-specific oxygen flux (JO2)] in isolated, permeabilized cardiac fiber bundles from mice modeled as in Figure 8a. Oxygen consumption was assessed in the presence of (A) glutamate and pyruvate (to assess complex I leak), (B) ADP (to assess complex I activity), and (C) rotenone (to assess complex II activity). See Methods section for complete description of experimental methods. Statistical comparisons were performed by one-way ANOVA followed by Tukey's test for multiple comparison testing between groups in A, and by Kruskal-Wallis test followed by Dunn's test for multiple comparison testing between groups in B and C. Statistically significant differences between groups are shown in panels; all other comparisons were $p>0.05$.
